# Galectin-3 and Myeloperoxidase May Monitor Cancer-Therapy-Related Cardiotoxicity? A Systematic Review and Meta-Analysis

**DOI:** 10.3390/biom12121788

**Published:** 2022-11-30

**Authors:** Yujuan Wu, Diansa Gao, Jinmin Xue, Zhong Zuo

**Affiliations:** 1 Chongqing Medical University, Chongqing 400016, China; 2 Department of Cardiology, The First Affiliated Hospital of Chongqing Medical University, Chongqing 400016, China; 3 Department of Oncology, The First Affiliated Hospital of Chongqing Medical University, Chongqing 400016, China

**Keywords:** **g**alectin-3, myeloperoxidase, biomarker, cardio-oncology, cardiotoxicity, cancer therapy

## Abstract

Galectin-3 and myeloperoxidase (MPO) are novel biomarkers in the field of cardio-oncology, but conflicting results have been reported. Hence, a meta-analysis was performed to assess the monitoring value of galectin-3 and MPO in cancer-therapy-related cardiotoxicity. PubMed, Cochrane, Web of Science, Embase, CNKI databases and ClinicalTrials.gov were queried. According to the predefined inclusion and exclusion criteria, eight studies with 1979 patients were included in this meta-analysis. The examination of the study’s heterogeneity (I^2^), quality assessment and statistical analysis were performed by two reviewers. No significant differences in galectin-3 levels were noted before and after treatment (WMD = −0.10, 90% CI −6.06–5.85, I^2^: 99%), and a weaker relationship was observed between galectin-3 evaluations and cancer-therapy-related cardiotoxicity (HR = 1.39, 90% CI 0.97–1.98, I^2^: 0%). However, MPO levels were increased in patients post-treatment (SMD = 0.58, 90% CI 0.35–0.80, I^2^: 56%), and an increased risk of cardiotoxicity was associated with early pre–post MPO assessments (HR = 1.16, 90% CI 1.02–1.32, I^2^: 21%). Surprisingly, the MPO levels were a more effective indicator of the response to tumor treatment compared with the TnI (SMD = 2.46, 90% CI −0.26–5.19, I^2^: 96%) and NT-proBNP levels (SMD = 1.08, 90% CI −0.82–2.98, I^2^: 96%). In conclusion, our meta-analysis suggests that MPO may rep-resent a potential biomarker for the early detection of cardiotoxicity in current cardio-oncology practice, but the monitoring value of galectin-3 requires further study.

## 1. Introduction

In recent years, cancer-therapy-related cardiotoxicity has induced life-threatening risks and long-term morbidity [1,2]; therefore, suitable knowledge, interpretation and management are needed and have to be placed within the context of the overall care of individual cancer patients [3]. Notably, heart failure is the most worrisome consequence of cardiotoxicity, and the goal of the cardio-oncology team is to provide timely diagnosis and optimal treatment [1,4,5]. Currently, the administration of radiotherapy, anthracycline chemotherapy, molecularly-targeted inhibitors and antibodies targeting immune checkpoints all negatively impact the patient’s cardiovascular system [6]. In particular, anthracyclines, including doxorubicin, epirubicin, idarubicin and daunorubicin, lead to cardiotoxicity through pathways of oxidative stress, calcium overload, lipid peroxidation and mitochondrial dysfunction [7].

Given that an impaired left ventricular ejection fraction (LVEF) may take weeks or months to become detectable by imaging [6], there is an increasing need for the early use of biomarkers, such as galectin-3 and myeloperoxidase (MPO), in order to detect the cardiotoxicity at a stage before it becomes irreversible [8]. However, as novel biomarkers, the roles of galectin-3 and MPO in the monitoring of cancer-therapy-related cardiotoxicity remain controversial. In this systematic review and meta-analysis, we assessed the value of galectin-3 and MPO in monitoring cardiotoxicity in cancer patients.

## 2. Methods

### 2.1. Search Strategy and Selection Criteria

**Figure 1:**
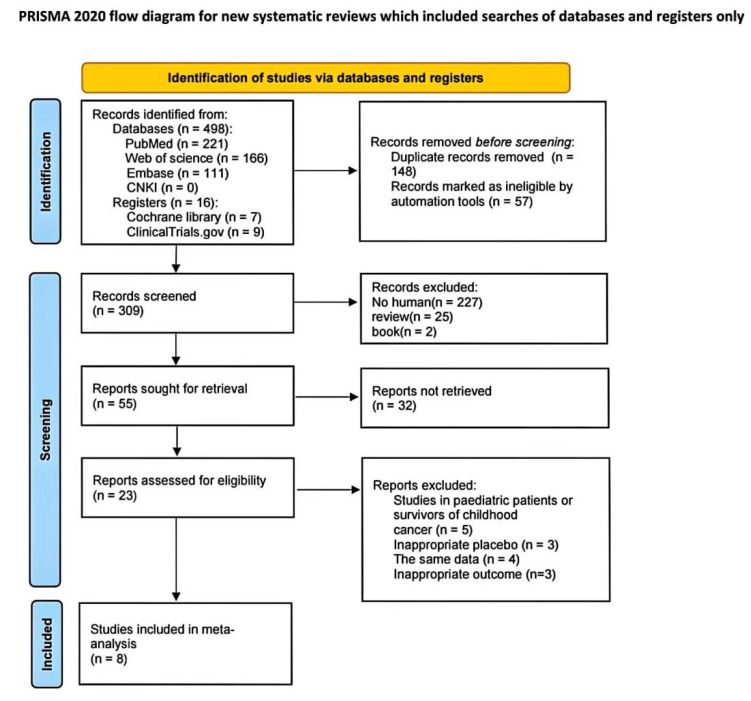
PRISMA 2020 flow diagram for new systematic reviews which included searches of databases and registers only.

This meta-analysis was conducted according to the Preferred Reporting Items for Systematic Reviews and Meta-Analysis (PRISMA) reporting guidelines [9]. The study was prospectively registered and accessed under PROSPERO (CRD42022354713). PubMed, Cochrane, Web of Science, Embase, CNKI databases and ClinicalTrials.gov were screened for studies that assessed galectin-3 and MPO levels during cancer therapy. The search included papers published from database inception until 26 August 2022. The following query terms were used to search for research articles: (Galectin 3[TIAB] OR myeloperoxidase, human[TIAB]) AND (Cardiotoxicity[TIAB]) AND (Neoplasms[TIAB]). Two reviewers independently screened the literature (Wu and Xue). A third individual was consulted in any cases of disagreement (Gao) (Figure 1).

### 2.2. Study Selection and Data Extraction

The following predetermined inclusion criteria were used: (i) patients with neoplasms on chemotherapy; (ii) galectin-3 and MPO levels were analyzed in peripheral blood; and (iii) randomized controlled trials, cohort studies (prospective observational studies) and case-control studies were included. The exclusion criteria were as follows: (i) studies with no mention of cardiac adverse events; (ii) duplicate published data; (iii) vague studies and those with key information that could not be extracted; (iv) unreasonable outcome indicators or outcome indicators that could not be combined; (v) reviews and books; and (vi) studies of pediatric patients or survivors of childhood cancers.

The purpose of this study was to identify any significant increases/decreases in Gal-3 and MPO in relation to cancer-therapy-related cardiotoxicity. The primary outcome was cardiotoxicity, which was defined according to recent guidelines as a reduction in the left ventricular ejection fraction (LVEF) of ≥5% to <55% with symptoms of heart failure, or an asymptomatic reduction in the LVEF of ≥10% to <55% [10,11]. The associated outcome was the relationship between the biomarkers and the risk of cardiotoxicity in the included papers, and hazard ratios (HRs) were reported. Data were extracted by two different researchers (Wu and Xue). Any discrepancy was evaluated by both researchers. If the discrepancy was not resolved, the third author (Gao) made the final decision. Data extracted included the year of publication, study type, demographic, cancer type, intervention (treatment modality), follow-up time, baseline LVEF, biomarker examined, outcome events and associated outcomes (Table 1). For data synthesis, the measured time, biomarker details (including mean and standard deviation (SD) of pre–post treatment and type of assay) and number of included patients or HR were recorded (Tables S1 and S2). The change of biomarkers was defined by the individual studies after the values of cancer therapy patients were compared to baseline values in patients serving as their own control. WebPlotDigitizer 4.5 was used to extract data from images in the study when appropriate biomarkers values were not present [12].

### 2.3. Quality Assessment

**Figure 2:**
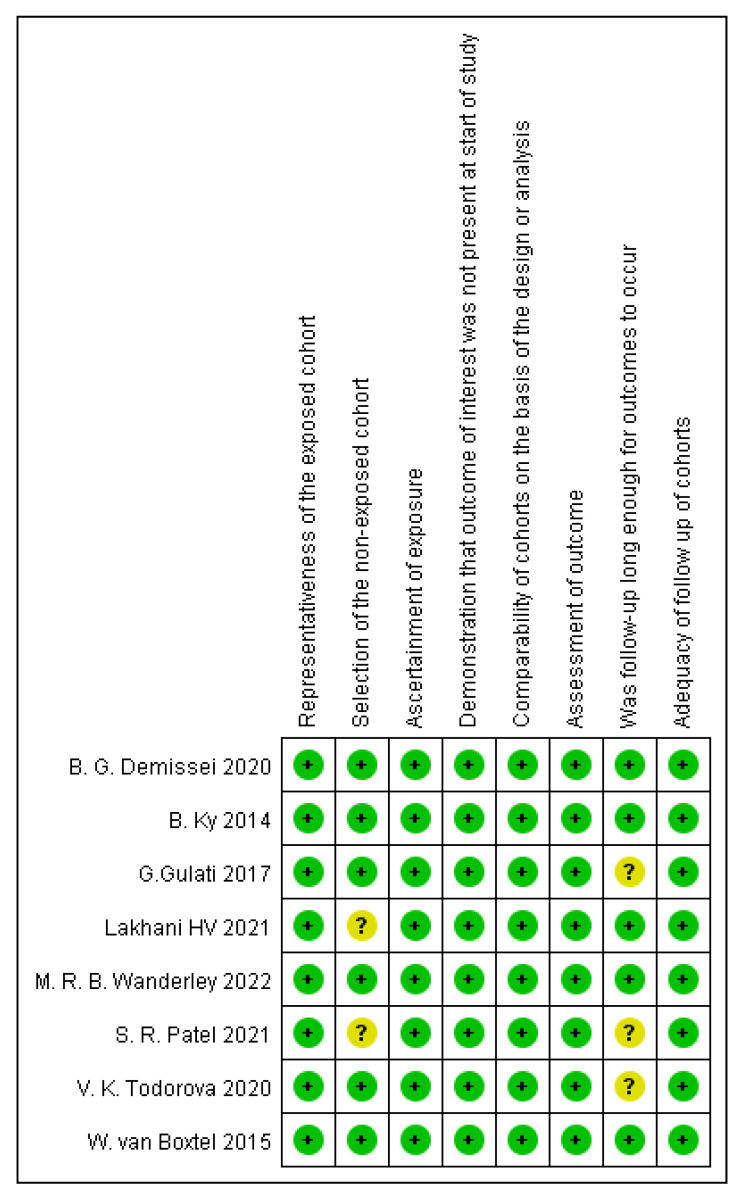
Quality assessment using the Newcastle-Ottawa Scale for cohort studies [13,14,15,16,17,18,19,20].

The Newcastle–Ottawa Scale (NOS) for cohort studies was used to assess the quality of the included studies, and the assessment was performed by two independent reviewers (Wu and Xue) [21] (Figure 2, Table S3). Studies were considered to be of a high quality if they had an NOS score ≥ 6 [22]. Review Manager (RevMan) version 5.4 was used to conduct quality assessments.

### 2.4. Statistical Analysis

Data are presented in several of the included studies as the median (interquartile range) rather than the mean ± SD. We performed data transformation prior to our data analysis based on the Cochrane Handbook for Systematic Reviews of Interventions [23]. We assessed heterogeneity using the inconsistency index (I^2^) and the χ^2^-based Q test; cutoffs of <30%, 30–59%, 60–75%, and >75% were used to suggest low, moderate, substantial, and considerable heterogeneity, respectively [24]. I^2^ > 50% indicated significant heterogeneity [25]. If the heterogeneity was significant (I^2^ > 50%), we used the random-effects model to conduct the statistical analysis; otherwise (I^2^ ≤ 50%), a fixed-effects model was used. For continuous variables, data are expressed as the weighted mean difference (WMD). The standardized mean difference (SMD) is utilized when the unit of measurement for data is not constant. The Inverse Variance method was used to calculate the WMD or SMD and its corresponding 90% confidence interval (CI). HRs were combined by using Generic inverse variance with 90% CI. We used subgroup analysis to determine the cause of any significant heterogeneity. Subgroup analyses were performed independently for each country, treatment modality and type of assay. Review Manager (RevMan) version 5.4 was used to calculate the data and perform subgroup analysis.

## 3. Results

### 3.1. Article Selection and Baseline Characteristics

The systematic search resulted in 309 potentially relevant articles. After the removal of review articles, animal studies and books, 55 studies remained following title and abstract screening (Figure 1). A total of eight research articles studying 1979 patients met the study criteria. All the included articles were prospective cohort studies. The majority of the research included studies from the United States, although data from the Netherlands, Norway, and Brazil were also included. The anticancer treatments were primarily anthracyclines, with doxorubicin being utilized in three articles [15,17,19]. The tumor types were almost all breast cancer, and only one of the studies focused on invasive ductal carcinoma. The mean age of the participants was 51.2 (±10.2) years, and the median follow-up time was 6 months (range: 0.5–44.4 months). The number of extractable outcome events is listed below (Table 1). HRs were reported in three publications to determine the connection between gal-3 and MPO, and the risk of cancer-therapy-related cardiotoxicity. In cases where the outcome event(s) occurred, the values of Gal-3 and MPO were only mentioned together in two publications. The remaining information on gal-3 was obtained from three articles, and that for MPO was obtained from two articles. Therefore, for the meta-analysis, five [14,15,17,18,19] and four [16,17,19,20] articles were considered to assess changes in gal-3 and MPO levels, respectively, before and after the cancer treatment (Tables S1 and S2).

### 3.2. Galectin-3 Does Not Increase in Response to Cancer Treatment

**Figure 3:**
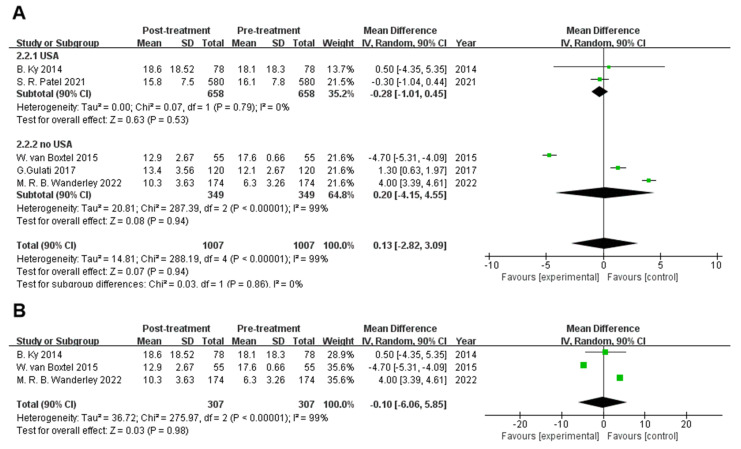

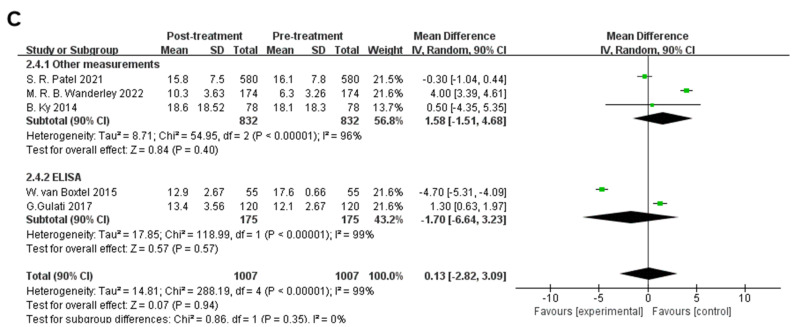
Overall and individual study estimates of the weighted mean difference of Galectin-3 are shown in patients receiving cancer treatment. Parallelogram boxes for weighted mean difference and horizontal lines represent 90% confidence interval (CI). Subgroup analyses were carried out independently for country (**A**), the presence of doxorubicin (**B**) and measurement (**C**) [14,15,17,18,19].

This analysis aimed to evaluate whether Galectin-3 levels are frequently elevated in response to cancer therapy. To evaluate the post-treatment compared to pre-treatment Galectin-3 levels, five studies (1007 patients) analyzing the absolute values were included. Summative weighted mean differences (WMD) revealed no significant difference in galectin-3 levels before and after the treatment [WMD 0.13 (90% confidence interval [CI] −2.82, 3.09), I^2^: 99%, *p* < 0.00001] (Figure 3).

Subgroup analyses were performed independently for the country, the presence or absence of doxorubicin and the type of assay due to significant heterogeneity (I^2^: 99%). Two studies [14,19] included data from the USA, whereas the remaining three used data from the Netherlands, Norway, and Brazil. As a result, groups of patients from the USA and not from the USA (Figure 3A) were included in the subgroup analysis. The USA population data showed no statistically significant difference in galectin-3 levels before and after treatment [USA WMD, −0.28 (90% CI −1.01, 0.45), I^2^: 0%, *p* = 0.79]; however, it is noteworthy that the USA group data were homogeneous. Population data from patients not living in the USA also showed no statistically significant difference [not living in the USA, WMD −0.20 (90% CI −4.15, 4.55), I^2^: 99%, *p* < 0.00001]. The subgroup analysis was performed based on the presence or absence of doxorubicin use. Doxorubicin was clearly used in three studies [15,17,19]; hence, a separate meta-analysis (Figure 3B) was performed for these three studies. The results indicate that galectin-3 levels did not statistically change before or after doxorubicin treatment [WMD −0.10 (90% CI −6.06, 5.85), I^2^: 99%, *p* < 0.00001]. Regarding the type of assay, two studies [15,18] used the same measurement technique, namely, enzyme-linked immunosorbent assay (ELISA), whereas the remaining investigations employed various approaches. Based on our subgroup analysis (Figure 3C), we discovered that the heterogeneity between the two studies employing the same methodology remained significant (I^2^: 96%, *p* < 0.00001), indicating that the use of various measuring techniques in this investigation was not the primary cause of heterogeneity.

In this meta-analysis, heterogeneity was unavoidable but was reduced using a multipathway analysis with a subgroup analysis based on the country where the study was performed. On the one hand, data heterogeneity persisted after subgroup analyses, so the use of subgroup analysis as a method to identify the reasons for the considerable heterogeneity was unsuccessful. On the other hand, as observed from the findings of the studies that were included, the outcomes differed. Specifically, the results of three studies [17,18,19] that were included revealed an increase in galectin-3 levels after treatment, and two studies [14,15] showed the opposite effect.

### 3.3. Galectin-3 Is Not Useful for the Monitoring of Cancer-Therapy-Related Cardiotoxicity

**Figure 4:**
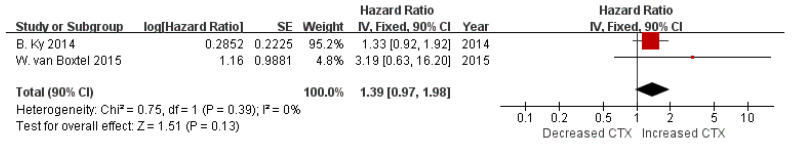
Cancer-therapy-related cardiotoxicity as measured by a standardized left ventricular ejection fraction decrease with no considerable Galectin-3 change. Parallelogram boxes for hazard ratio, and horizontal lines represent 90% confidence interval (CI) [15,19].

The purpose of this analysis was to assess the association between galectin-3 change and the risk of cardiotoxicity. A total of two studies [15,19] (133 patients) were included in the assessment of pre–post galectin-3 levels in relation to cancer-therapy-related cardiotoxicity with an HR of 1.39 (90% CI 0.97, 1.98) (I^2^: 0%, *p* = 0.39) (Figure 4). The heterogeneity is low.

### 3.4. MPO Increases in Response to Cancer Treatment

**Figure 5:**
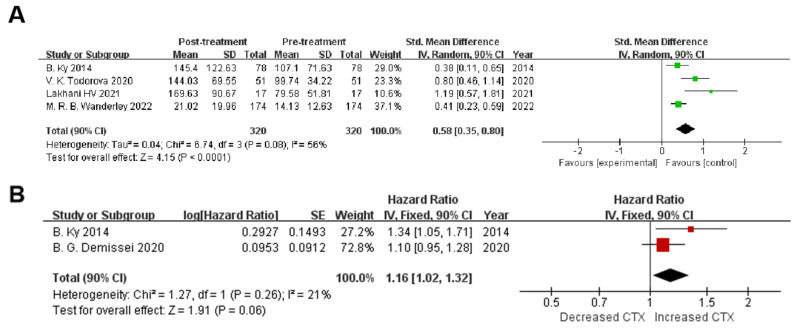
(**A**) Overall and individual study estimates of the standardized mean difference of MPO are shown in patients receiving cancer treatment. Parallelogram boxes for standardized mean difference, and horizontal lines represent 90% confidence interval (CI). (**B**) Cancer-therapy-related cardiotoxicity as measured by a standardized left ventricular ejection fraction decrease with significant MPO change. Parallelogram boxes for hazard ratio, and horizontal lines represent 90% confidence interval (CI) [13,16,17,19,20].

To assess any changes in MPO levels in response to cancer therapy, four studies (320 patients) were included in the analysis of absolute values. MPO levels were measured in all patients three months prior to and three months following therapy, and in 51 patients one-half month following their treatment. Mean MPO levels were higher in patients upon the administration of cancer therapy [SMD 0.58 (90% CI 0.35, 0.80), I^2^: 56%, *p* = 0.08] (Figure 5A). The analysis showed moderate heterogeneity (I^2^: 56%), possibly due to variations in the methodology of biomarker assessment.

### 3.5. MPO for the Monitoring of Cancer-Therapy-Related Cardiotoxicity

The following meta-analysis aimed to determine the role of MPO in the monitoring of cancer-therapy-related cardiotoxicity. Two studies [13,19] (401 patients) were included. MPO levels were all assessed pre-treatment and three months post-treatment. The results indicated that early pre–post MPO evaluations were related to an increased risk of cardiotoxicity [HR 1.16 (90% CI 1.02,1.32), I^2^: 21%, *p* = 0.26] (Figure 5B). The heterogeneity is lower (I^2^: 21%) than in the analysis of changes of MPO levels in response to cancer therapy.

### 3.6. MPO Levels Increased Earlier Than TnI and Nt-proBNP Levels in Response to Cancer Treatment

We extracted data on TnI and NT-proBNP levels (mean, SD and sample size) simultaneously before and after treatment from the four studies that reported data on MPO levels for further meta-analysis to assess the value of early MPO levels as an indicator of cancer-therapy-related cardiotoxicity and to assess the heterogeneity of the studies [16,19]. No obvious differences in TnI (Figure S1A) and NT-proBNP (Figure S1B) levels were noted before and after treatment [TnI, SMD 2.46 (90% CI −0.26, 5.19), I^2^: 96%, *p* < 0.00001; NT-proBNP, SMD 1.08 (90% CI −0.82, 2.98), I^2^: 96%, *p* < 0.00001]. Thus, MPO levels are more sensitive to cancer treatment than TnI and NT-proBNP levels. Although the analysis revealed considerable heterogeneity for TnI (I^2^ = 96%) and for NT-proBNP (I^2^ = 96%), subgroup analyses could not be performed given the small number of studies.

## 4. Discussion

The present meta-analysis reveals that MPO is a useful biomarker for the early detection of cancer-therapy-related cardiotoxicity, whereas galectin-3 is not. Unfortunately, Galectin-3 has not been evaluated previously in relation to anthracycline treatment.

### 4.1. Biomarker Pathway

Galectin-3 is a member of the beta galactoside binding lectin family involved in cardiac fibrosis, heart failure (HF) and atherosclerosis [26]. Galectin-3, which is secreted by macrophages, enhances cardiac fibrosis and influences the immune response, leading to pernicious cardiac remodeling. High levels of this biomarker were associated with an increased risk of death in HF patients [27]. Conflicting results have been reported regarding the ability of Galectin-3 to predict cancer-therapy-related cardiotoxicity. The absence of a difference in pre-treatment and post-treatment galectin-3 levels, and the weaker relationship between galectin-3 evaluations and the risk of cardiotoxicity, suggest that galectin-3 is likely not a useful biomarker of cancer-therapy-related cardiotoxicity. On the one hand, it is probable that the use of galectin-3 to diagnose cardiovascular illness in cancer survivors in clinical practice may be impacted by the overlap between the pathophysiology of cardiovascular disease and cancer, including inflammation and cell proliferation [28]. On the other hand, the unsatisfactory results of galectin-3 as a biomarker for cancer-therapy-related cardiotoxicity in humans may be related to the inability of echocardiography to accurately assess myocardial fibrosis [29]. Therefore, additional multicenter studies with substantial sample sizes are required to corroborate the assessment of the predictive potential of galectin-3 in cancer-therapy-related cardiotoxicity.

**Figure 6:**
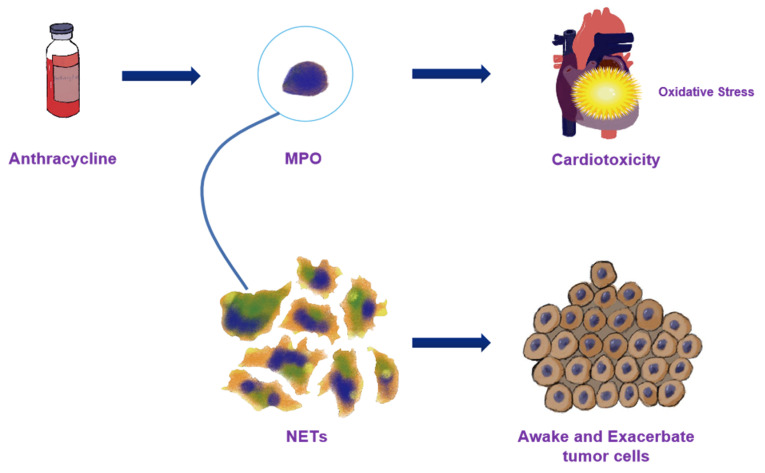
The potential mechanism of MPO increasing after cancer therapy. MPO: myeloperoxidase; NETs: neutrophil extracellular traps.

MPO is an enzyme primarily produced by neutrophils that plays a key role in the pathophysiology of coronary artery disease, congestive heart failure, arterial hypertension, and other cardiovascular diseases [30]. In patients with ACS, MPO serum levels strongly predict an increased risk of subsequent cardiovascular events [31]. Elevated MPO levels are associated with a poor prognosis and a high risk of cardiovascular disease [30]. According to the findings of our meta-analysis, MPO levels were noticeably increased in patients following cancer treatment. As a proatherogenic enzyme, MPO can lead to inflammation and oxidative stress [32]. Oxidative stress is thought to be one of the main mechanisms of doxorubicin-induced cardiotoxicity [33]. As a result, anthracyclines might exacerbate oxidative stress by overactivating MPO in addition to anticancer therapy, which significantly increases the risk of cardiovascular events. Therefore, MPO is a potential biomarker of cancer-therapy-related cardiotoxicity. However, it is worth noting that, MPO is a component of neutrophil extracellular traps (NETs), a crucial structure with which neutrophils eliminate infections or other endogenous or external elements [34]. It has been suggested that NETs awaken dormant cancer cells, play a key regulatory role in the tumor microenvironment, and exacerbate tumor aggressiveness by enhancing cancer migration and invasion capacity [35]. Thus, elevated MPO levels could indicate tumor progression. The tumor itself may also be to blame for the increase in MPO levels following anticancer therapy in addition to cardiotoxicity (Figure 6). However, in this study, we directly showed that elevated MPO levels are related to the risk of cardiotoxicity (HR 1.16, 90% CI 1.02–1.32). Given the limited sample size of this meta-analysis, it is hoped that future research will further explore the mechanism by which MPO levels are increased in cardio-oncology.

### 4.2. Strengths and Opportunities for Future Research

The American Society of Clinical Oncology guidelines recommend monitoring the levels of troponins and BNP/NT-proBNP in patients receiving anthracyclines at risk for cardiotoxicity during and after cancer treatment [36]. However, the findings of several clinical trials [19,37,38,39] have been contradictory, and no correlation with cardiotoxicity has been shown. With regard to the potential limitations of established cardiac biomarkers in cancer-therapy-related cardiotoxicity, galectin-3 and MPO are currently under investigation for their use in the early detection of cardiotoxicity [8,40]. At the initial stage of cancer treatment, the median measured time was 3 months (range: 0.5–3 months), and the meta-analysis results reveal that MPO levels serve as an earlier marker of cardiotoxicity compared with TnI and NT-proBNP levels. This meta-analysis innovatively assessed the value of new biomarkers (galectin-3, MPO) in the monitoring of cardiotoxicity associated with antitumor therapy and explained the mechanisms involved. We demonstrate directly that increased MPO levels are associated with a risk of cardiotoxicity. However, the roles of these proteins in cardiovascular and neoplastic events are still not completely understood. We tried to further explore whether the tumor is progressive in patients with increased MPO; unfortunately, no prognostic data of tumors were extracted. Current studies mostly separate cancer-therapy-related cardiotoxicity from tumor progression. Future clinical studies can assess the combination of multiple biomarkers, such as cardiac markers (troponins and BNP/NT-proBNP) and other circulating NET markers (neutrophil elastase) [35], to better understand the relationship between tumor progression and cardiotoxicity. The identification of MPO for the specific monitoring of cardiotoxic mechanisms in cancer therapy may further enhance the detection of cancer-therapy-related cardiotoxicity.

### 4.3. Limitations

Our meta-analysis has some limitations that should be mentioned. First, the data heterogeneity is attributed to the use of different patient populations and study types; different reagents, test kits and laboratory equipment to assess biomarkers and concentrations; and different echocardiographic devices. In addition, considerable heterogeneity might have confounded the summative galectin-3 assay (I^2^ = 99%), whereas no heterogeneity was found for the subgroup analyses based on the country of study and assay used (I^2^ = 0%). The data included studies from many countries and were quite heterogeneous, and this heterogeneity could be attributed to ethnic variances in the study population. Consequently, more information from other countries and ethnic groups is required to fully investigate the role of galectin-3 in the monitoring of cardiotoxicity during anticancer therapy. Second, due to the current lack of clinical data, the number of studies included in this analysis was small. In addition, only one tumor type was assessed in each study, and the treatment modalities were limited. Additional information regarding the measurement of galectin-3 and MPO levels in various tumor types and following various treatment regimens is required to further improve the meta-analysis. Finally, we did not further discuss whether the earlier detection of cardiotoxicity could prevent irreversible cardiotoxicity.

## 5. Conclusions

The importance of detecting cardiotoxicity before there is a decline in EF underscores the importance of the use of biomarkers as tools for the early detection of cardiotoxicity in current cardio-oncology practice. The present meta-analysis highlights MPO as a promising biomarker of cancer-therapy-related cardiotoxicity, whereas the available evidence does not yet consistently support a beneficial role for galectin-3, potentially due to significant heterogeneity. In the present systematic review and meta-analysis, we found that novel biomarkers have strong potential to identify patients undergoing cancer treatment who are at high risk for cardiotoxicity, and provide future research directions for the field of cardio-oncology.

## Figures and Tables

**Table 1:** Study Characteristics. Values are expressed as mean ± SD or median [interquartile range] for continuous variables. LVEF: Left Ventricular Ejection Fraction; gal-3: galectin-3; MPO: myeloperoxidase. +: The main outcome event occurred, but the number of events was not extracted; —: No accurate data were extracted. ①②: Age distribution of different interventions.

Study Characteristics												
Author	Year	Sample Size	Study Type	Country	Age (Mean; Interquartile Range/SD)	Cancer Type	Intervention (Treatment Modality)	Follow-Up (Months)	Baseline LVEF % (Mean; Interquartile Range/SD)	Biomarker Examined	Outcome Events (n)	Associated Outcome (HR)
B. G. Demissei et al. [13]	2020	323	Prospective	USA	48 (41–57)	breast cancer	doxorubicin + Trastuzumab	44.4	53 (51–56)%	MPO	49	MPO: 1.1 (0.92–1.31)
S. R. Patel et al. [14]	2021	1160	Prospective	USA	52.2 ± 9.6	breast Cancer	Anthracyclines	32.4	—	gal-3	+	—
W. van Boxtel et al. [15]	2015	55	Prospective	Netherlands	52.8 ± 8.5	breast cancer	docetaxel + doxorubicin + cyclophosphamide (TAC)	12	—	gal-3	5	gal-3: 3.19 (0.46, 22.20)
Lakhani HV et al. [16]	2021	17	Prospective	USA	57.6 ± 4.5	invasive ductal carcinoma	Anthracyclines	6	62.9 ± 1.2%	MPO	4	—
M. R. B. Wanderley et al. [17]	2022	174	Prospective	Brazil	50.4 ± 9.5	breast Cancer	Anthracyclines	6	—	gal-3, MPO	26	—
G.Gulati et al. [18]	2017	121	Prospective	Norway	① Epirubicin = 400 mg/m^2^: 51.0 (42.0, 59.0) n = 27 ② Epirubicin < 400 mg/m^2^: 48.5 (43.8, 58.0) n = 94	breast cancer	Anthracyclines	3.2	63.3 ± 4.0%	gal-3	1	—
B. Ky et al. [19]	2014	78	Prospective	USA	50.0 (42.0–56.8)	breast cancer	Anthracyclines	3	64 (61–68)%	gal-3, MPO	+	gal-3: 1.33 (0.86–2.05) MPO: 1.34 (1.00–1.80)
V. K. Todorova et al. [20]	2020	51	Prospective	USA	52.2 ± 11.5	breast cancer	doxorubicin	0.5	64.5 ± 7.0%	MPO	+	—

## Data Availability

Data collected for this study are available upon request. Queries should be directed to the corresponding author: 202761@hospital.cqmu.edu.cn.

## Supplementary Materials

The following supporting information can be downloaded at: https://www.mdpi.com/article/10.3390/biom12121788/s1.
