# Supplementary material for: Galectin-3 and Myeloperoxidase May Monitor Cancer-Therapy-Related Cardiotoxicity? A Systematic Review and Meta-Analysis
Source: Biomolecules. 2022 Nov 30;12(12):1788. doi: 10.3390/biom12121788 (PMC9775944; doi:10.3390/biom12121788)
Supplement: Supplementary file 1 [file biomolecules-12-01788-s001.zip › biomolecules-2017606-supplementary.pdf]

Studies included in Gal-3 meta-analysis							
Author	Year	Sample size	Measured time(months)	Gal-3 values(ng/ml)		Measurement	Associated outcome(HR)
				pre-treatment	post-treatment		
B. Ky	2014	78	3	18.1±18.3	18.6±18.52	Microparticle chemiluminescence immunoassay(MCIA)	1.33 (0.86-2.05)
W. van Boxtel	2015	55	12	17.6±0.66☆	12.9(11.2，14.8)	Enzyme-linked immunosorbent assay(ELISA)	3.19 (0.46, 22.20)
G.Gulati	2017	120	3	12.1(10.4, 14.0)	13.4(11.2, 16.0)	ELISA	—
S. R. Patel	2021	1160	3	16.1±7.8	15.8±7.5	Enzyme-linked fluorescent assay(ELFA)	—
M. R. B. Wanderley	2022	174	3	6.3(5.2，9.6)	10.3(8.2，13.1)	Milliplex map kit	—

Table S1: Studies included in Gal-3 meta-analysis.Values are expressed as mean±SD or median [interquartile range] for continuous variables. ⭐: The standard value of this index in the paper was used since the data from the control group was not given in the paper. —:

No accurate data was extracted.

Studies included in MPO meta-analysis							
Author	Year	Sample size	Measured time(months)	MPO values(ng/ml)		Measurement	Associated outcome(HR)
				pre-treatment	post-treatment		
B. Ky	2014	78	3	156.3±159.1 (pmol/L)	145.4±122.63☆ (pmol/L)	Microparticle chemiluminescence immunoassay(MCIA)	1.34 (1.00-1.80)
V. K. Todorova	2020	51	0.5	99.74±34.22	144.03±69.55	Enzyme-linked immunosorbent assay(ELISA)	—
Lakhani HV	2021	17	3	79.58±51.81☆	169.63±90.67☆	Enzyme-linked fluorescent assay(ELFA)	—
M. R. B. Wanderley	2022	174	3	8.9 (7.9, 24.8)	12.94 (11.1, 37.8)	Milliplex map kit	—
B. G. Demissei	2020	323	3	—	—	ELISA	1.1 (0.92-1.31)

Table S2: Studies included in MPO meta-analysis.Values are expressed as mean±SD or median [interquartile range] for continuous

variables.★:This was extracted from the article image using WebPlotDigitizer 4.5. —: No accurate data was extracted.

Study	Selection				Comparability	Outcome			Total scores (9 scores)
	Representativeness of the exposed cohort	Selection of the non-exposed cohort	Ascertainment of exposure	Demonstration that outcome of interest was not present at start of study	Comparability of cohorts on the basis of the design or analysis	Assessment of outcome	Was follow-up long enough for outcomes to occur	Adequacy of follow up of cohorts	
B. Ky, 2014	★	★	★	★	★★	★	★	★	9
W. van Boxtel, 2015	★	★	★	★	★★	★	★	★	9
G.Gulati, 2017	★	★	★	★	★★	★		★	8
B. G. Demissei, 2020	★	★	★	★	★★	★	★	★	9
V. K. Todorova, 2020	★	★	★	★	★★	★		★	8
S. R. Patel, 2021	★		★	★	★★	★		★	7
Lakhani HV, 2021	★		★	★	★★	★	★	★	8

M. R. B. Wanderley, 2022	☆	☆	☆	☆	☆☆	☆	☆	☆	9

Table S3: Quality assessment using the Newcastle-Ottawa Scale for cohort studies.

A

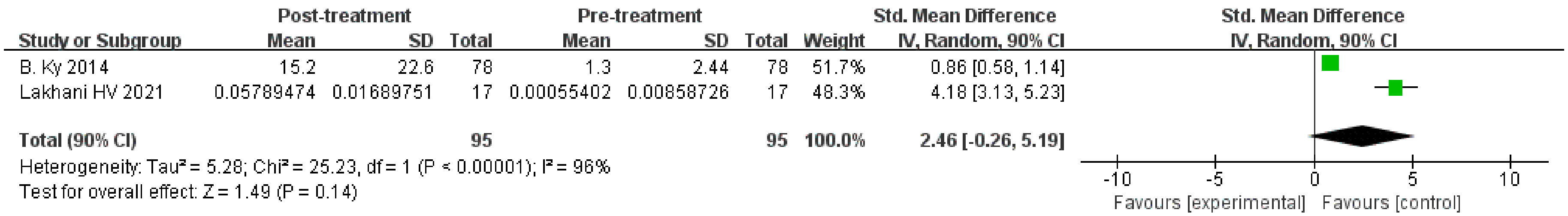

B

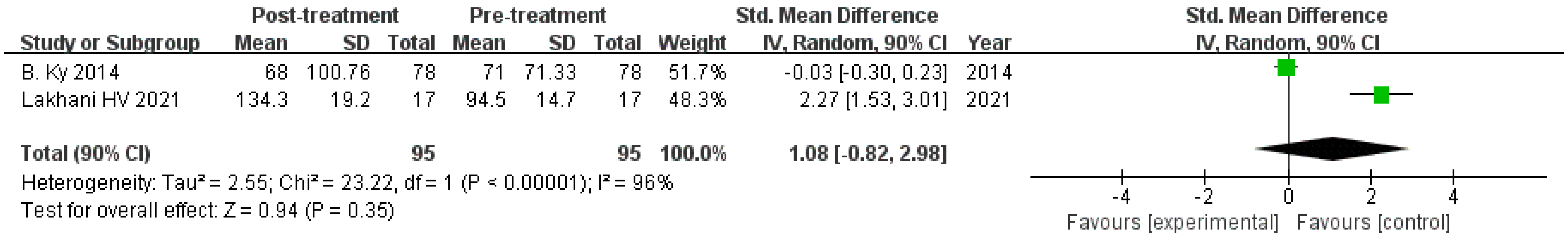

<u>Figure S1</u> Overall and individual study estimates of the standardized mean difference of TnI and NT-proBNP are shown in patients receiving cancer treatment. Parallelogram boxes for standardized mean difference, and horizontal lines represent 90% confidence interval (CI). (A) TnI; (B) NT-proBNP.
